# Pan-cancer investigation reveals mechanistic insights of planar cell polarity gene *Fuz* in carcinogenesis

**DOI:** 10.18632/aging.202582

**Published:** 2021-02-26

**Authors:** Zhefan Stephen Chen, Xiao Lin, Ting-Fung Chan, Ho Yin Edwin Chan

**Affiliations:** 1 Nexus of Rare Neurodegenerative Diseases, School of Life Sciences, Faculty of Science, The Chinese University of Hong Kong, Shatin, N.T., Hong Kong SAR, China; 2 School of Life Sciences, Faculty of Science, The Chinese University of Hong Kong, Shatin, N.T., Hong Kong SAR, China; 3 Gerald Choa Neuroscience Centre, The Chinese University of Hong Kong, Shatin, N.T., Hong Kong SAR, China

**Keywords:** planar cell polarity, pan-cancer analysis, gene set enrichment analysis, DNA methylation, genomic alteration

## Abstract

The fuzzy planar cell polarity protein (Fuz) is an effector component of the planar cell polarity (PCP) signaling. Together with other core and effector proteins, the PCP pathway controls polarized cell movements. Fuz was also reported as a negative regulator of cell survival. In this study, we performed a pan-cancer survey to demonstrate the role of Fuz in multiple types of cancer. In head-neck squamous cell carcinoma and lung adenocarcinoma tumor samples, a reduction of *Fuz* transcript expression was detected. This coincides with the poor overall survival probabilities of these patients. We further showed that *Fuz* promoter hypermethylation contributes to its transcriptional downregulation. Meanwhile, we also identified a relatively higher mutation frequency at the 404^th^ arginine amino acid residue in the coding sequence of *Fuz* locus, and further demonstrated that mutant Fuz proteins perturb the pro-apoptotic function of Fuz. In summary, our study unveiled an intriguing relationship between *Fuz* dysregulation and cancer prognosis, and further provides mechanistic insights of Fuz’s involvement in carcinogenesis.

## INTRODUCTION

The planar cell polarity (PCP) pathway is an evolutionarily conserved signaling axis that organizes the polarized movements of cells within a planar plane to achieve tissue patterning and morphogenesis [1]. Initial studies on the molecular functions of PCP pathway emphasize the relationship between PCP signaling and mammalian embryonic development, as exemplified by the fact that genetic alterations in PCP genes lead to severe neurodevelopmental deficits [2–4]. In addition to developmental defects, the PCP pathway also takes part in human disorders, including Alzheimer’s disease [1] and cancer [5, 6].

Two subsets of genes, including PCP core and effector genes, contribute to the signal transduction of the PCP pathway [7]. It is generally accepted that PCP effectors function genetically downstream of PCP core genes [8]. However, a few studies also indicate that the cellular functions of PCP core proteins could be reversely influenced by the PCP effectors, suggesting a more complex regulatory network between PCP core and effector genes in PCP signaling [9–11]. Cancer describes a broad range of diseases characterized by sustained cell proliferation and metastasis triggered by cell invasion to nearby tissues and organs [12, 13]. Several key signaling pathways, including those essential for embryonic neurodevelopment, are reported to function during tumorigenesis as well [14–16]. This indicates that some molecular mechanisms in human neurological disorders and cancer could be mutually inclusive. Recent research further unveiled the role of PCP signaling in cancer malignancy [17] and cancer cell dissemination [18, 19]. Moreover, upregulation of PCP core gene expression, *Prickle1* and *Vangl2*, inhibits neuroblastoma cell overproliferation, and corresponds to better survival probabilities in cancer patients [20].

Fuzzy planar cell polarity protein (Fuz) is categorized as a PCP effector. Similar to the other PCP core and effector players, the molecular function of Fuz was initially studied in mammalian embryonic development. The *Fuz* knockout (*Fuz*^-/-^) mice showed severe developmental retardation accompanied by the impairments in ciliogenesis and several essential developmental pathways, in particular, the Hedgehog signaling axis [10, 21]. At the cellular level, Fuz protein localizes to the basal body and ciliary axoneme to support cilia outgrowth [11]. Meanwhile, Fuz interacts to mediate the ciliary trafficking of Dishevelled (Dvl) protein, an intermediator responsible for signal transduction in Wnt pathways, suggesting a potential function of Fuz in Wnt signaling [11]. As observed in the embryonic fibroblasts isolated from *Fuz*^-/-^ mice, canonical Wnt/β-catenin signaling pathway was hyperactivated as demonstrated by the accumulation of nuclear β-catenin protein [11]. The expression of a group of β-catenin-targeted genes was also shown to be upregulated, which in turn causes an enhanced cell proliferation in *Fuz*^-/-^ mouse embryos [22]. In our previous study, we reported a novel pro-apoptotic function of the Fuz protein. When overexpressed, Fuz stimulates Dvl-Rac1-MAPK-caspase signaling cascade to trigger cell apoptosis [23]. Taken together, these findings support a crucial role of Fuz in mediating cell survival.

A previous study showed that overexpression of Fuz protein suppresses the growth of liver cancer cell line HEP1 *in vitro*, as well as in a mouse xenograft model [24]. Given the negative regulatory function of Fuz in controlling cell viability, we performed a pan-cancer survey to investigate the potential role of *Fuz* in multiple types of cancer. *Fuz* mRNA level was found downregulated in head-neck squamous cell carcinoma (HNSC) and lung adenocarcinoma (LUAD) tumor samples, and such downregulation contributes to a reduction of the overall survival probabilities in patients. Moreover, we demonstrated a tight correlation between *Fuz* transcription and *Fuz* DNA methylation level, and further showed that reduction of *Fuz* mRNA level in HNSC and LUAD tumor samples was achieved via hypermethylation of two independent CpG sites within *Fuz* promoter region. We also identified coding sequence alterations in the *Fuz* locus across different cancer types and highlighted a relatively higher mutation frequency at 404^th^ arginine residue. Functional analyses demonstrated that the pro-apoptotic capability of mutant Fuz protein is attenuated. In summary, this study is the first report to provide an in-depth investigation of Fuz in multiple types of cancer, and further demonstrates the effects of *Fuz* promoter DNA methylation and coding sequence alterations in cancer.

## RESULTS

### Investigation of prognostic value of *Fuz* in multiple cancer types

**Figure 1:**
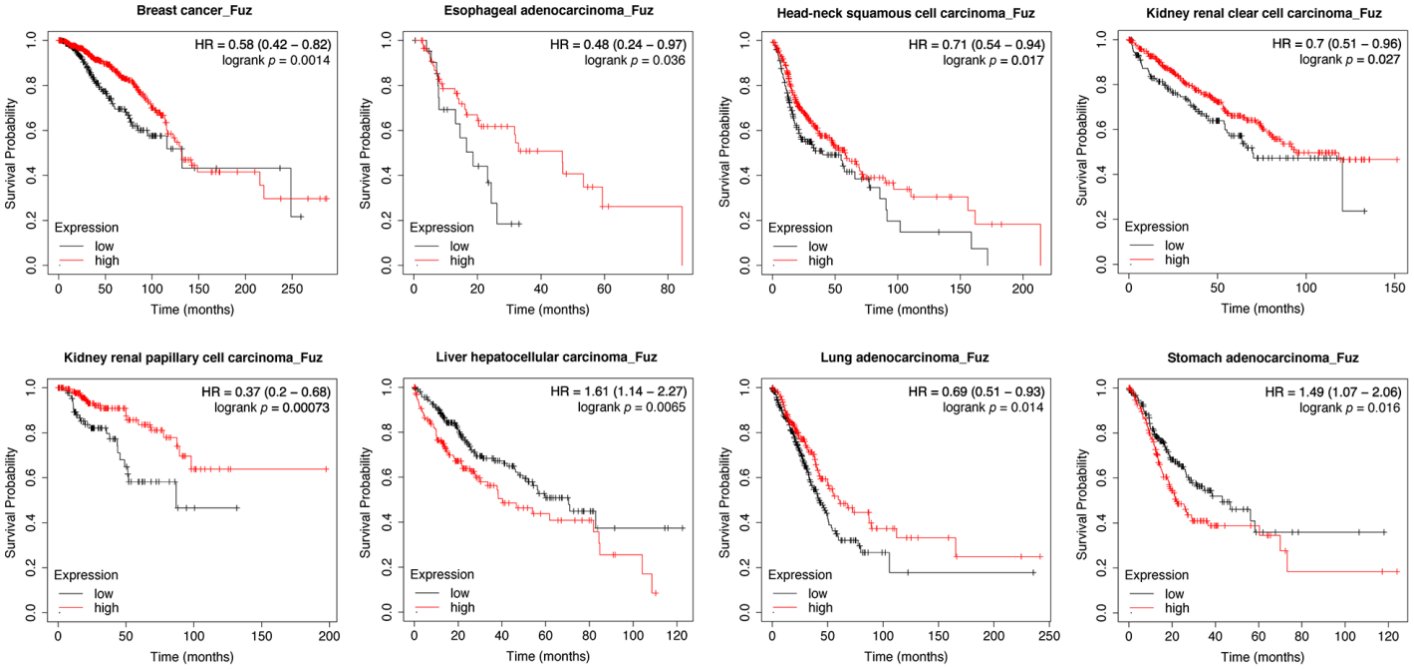
**Evaluation of the prognostic significance of Fuz mRNA expression in different cancer types.** Kaplan-Meier pan-cancer survival analysis was performed to evaluate the relationship between *Fuz* mRNA level and overall survival probabilities in 21 different types of cancer. Low level of *Fuz* expression was found associated with poor prognosis in BRCA, ESCA, HNSC, KIRC, KIRP and LUAD patients, whilst high level of *Fuz* expression was found associated with poor prognosis in LIHC and STAD patients.

We initially used Kaplan-Meier plotter (https://kmplot.com/analysis/) to assess the prognostic significance of *Fuz* mRNA expression in 21 different types of cancer. As shown in the Kaplan-Meier curves, *Fuz* expression was found to be significantly associated with the overall survival (OS) of patients from 8 types of cancer (Figure 1). In liver hepatocellular carcinoma (LIHC) and stomach adenocarcinoma (STAD), increased *Fuz* transcript level was shown to have poor OS in patients. However, in the remaining 6 types, i.e. breast cancer (BRCA), esophageal adenocarcinoma (ESCA), head-neck squamous cell carcinoma (HNSC), kidney renal clear cell carcinoma (KIRC), kidney renal papillary cell carcinoma (KIRP) and lung adenocarcinoma (LUAD), a reduced expression of *Fuz* was expected to contribute to decreased survival probabilities (Figure 1). The prognostic significance of *Fuz* mRNA expression was further assessed in different subtypes of breast cancer. Reduced level of *Fuz* was found to be significantly associated with poor OS in luminal A and HER2+ breast cancer patients (Supplementary Figure 1A). In addition, the prognostic significance of *Fuz* in breast cancer (Supplementary Figure 1B), lung cancer (Supplementary Figure 1C) and gastric cancer (Supplementary Figure 1D) was further supported by additional Gene Expression Omnibus (GEO) datasets.

The expression level of *Fuz*, together with other clinicopathological variables (gender, race, ethnicity, primary diagnosis, tumor stage, age at diagnosis), were analyzed and included in multivariate statistics. As summarized in Supplementary Table 1, decreased level of *Fuz* was an independent predictor of worse OS of BRCA (HR, 0.9996; 95% Cl, 0.9993-1.000; *p* value, 0.0106) and KIRP (HR, 0.9988; 95% Cl, 0.9981-1.000; *p* value, 0.00241) patients, whereas high expression of *Fuz* was independently associated with worse OS of STAD (HR, 1.001; 95% Cl, 1.0002-1.001; *p* value, 0.00257) patients.

### Examination of *Fuz* transcript level in multiple cancer types

**Figure 2:**
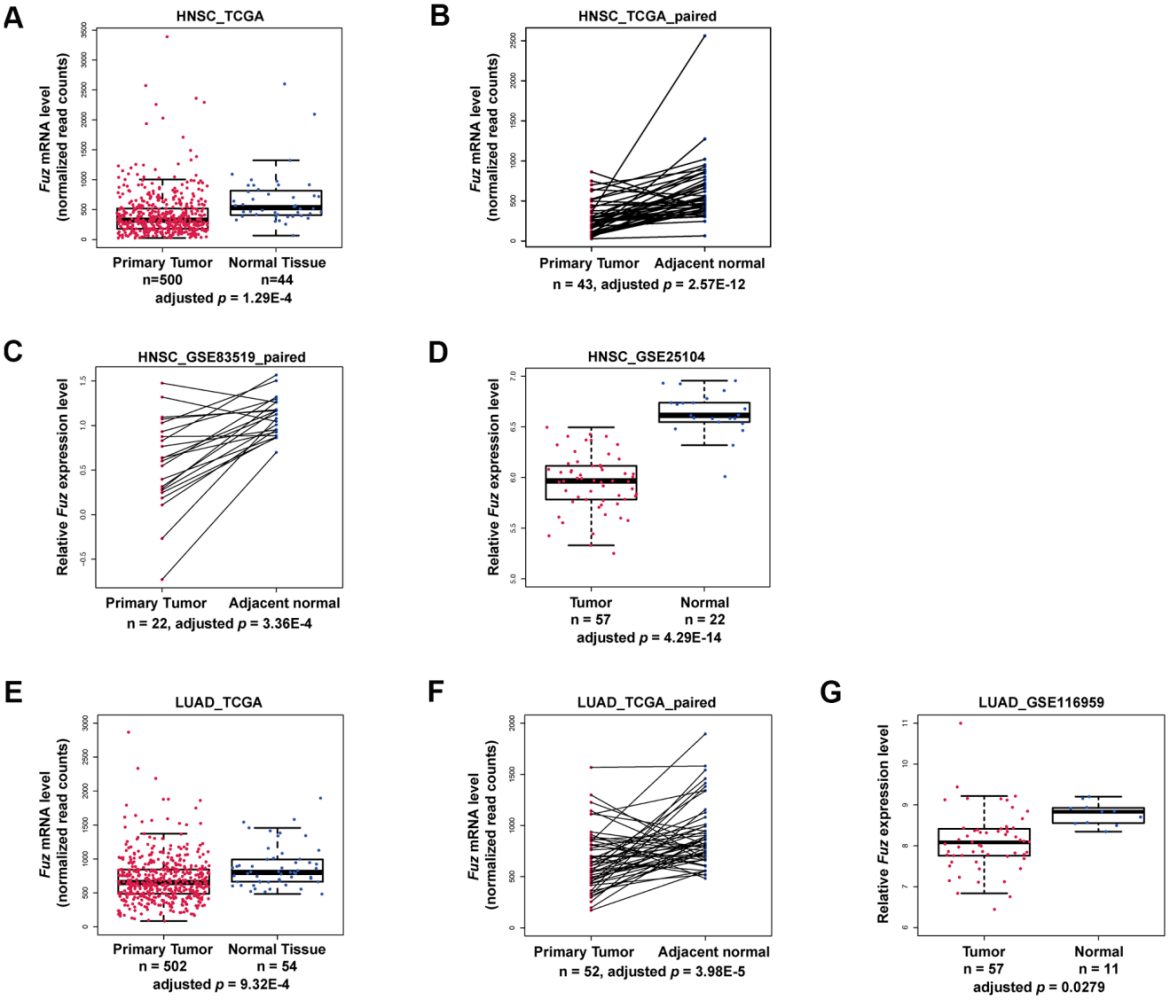
**Fuz mRNA level was downregulated in HNSC and LUAD patients.** (**A**–**D**) The expression of *Fuz* was found significantly downregulated in tumor tissues from HNSC patients. The datasets used for analysis were from TCGA, TCGA paired samples, GSE83519 and GSE25104, respectively. (**E**–**G**) The expression of *Fuz* was found significantly downregulated in tumor tissues from LUAD patients. The datasets used for analysis were from TCGA, TCGA paired samples and GSE116959.

Based on our patient survival analysis (Figure 1 and Supplementary Figure 1), we next determined if *Fuz* mRNA expression was altered in primary tumor tissues. Transcriptomic profiling data mining from The Cancer Genome Atlas (TCGA) data set repository (https://portal.gdc.cancer.gov/repository) was used to evaluate *Fuz* mRNA level in all primary tumor and solid normal tissues. Moreover, paired samples, with each pair comprises tumor and adjacent non-tumor samples from the same patient, were also included to provide more convincing evaluation of *Fuz* mRNA alterations. In HNSC (Figure 2A, 2B), LUAD (Figure 2E, 2F), LIHC (Supplementary Figure 2E) and STAD (Supplementary Figure 2F), *Fuz* expression was significantly downregulated in primary tumor tissues, and such downregulation was further observed in their paired samples. Downregulation of *Fuz* in HNSC (Figure 2C, 2D), LUAD (Figure 2G) and LIHC (Supplementary Figure 3) tumor samples was further validated in additional GEO datasets. In BRCA, although *Fuz* expression was found significantly altered in primary tumor tissues, no significant alteration was identified between tumor and paired non-tumor samples (Supplementary Figure 2A). No significant change in *Fuz* expression was observed in ESCA (Supplementary Figure 2B), KIRC (Supplementary Figure 2C) and KIRP (Supplementary Figure 2D) patient samples.

In line with our survival analysis, lowered expression of *Fuz* detected in HNSC and LUAD tumor samples (Figure 2) coincides with poor OS in patients (Figure 1). We thus decided to focus on HNSC and LUAD in our subsequent studies.

### Gene set enrichment analysis of genes from HNSC tumor samples with low *Fuz* expression

**Figure 3:**
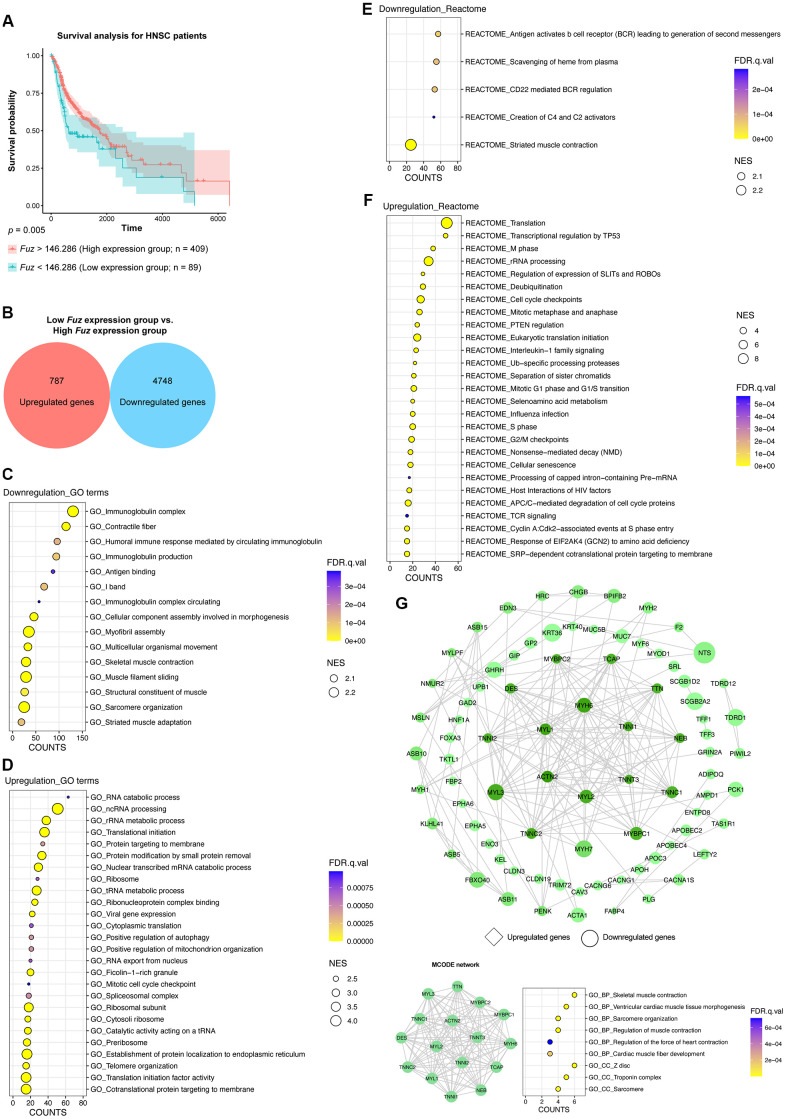
**The gene set enrichment and PPI analyses of dysregulated genes from HNSC tumor samples with low Fuz expression.** (**A**) The HNSC primary tumor tissues were divided into high *Fuz* expression and low *Fuz* expression groups based on the patient survival probabilities. (**B**) The number of upregulated and downregulated genes were calculated from HNSC primary tumor tissues with low *Fuz* expression. (**C**, **D**) The GO enrichment analysis demonstrated downregulated (**C**) and upregulated (**D**) genes-associated gene ontology terms in low *Fuz* expression group. (**E**, **F**) The Reactome pathway enrichment analysis demonstrated the downregulated (**E**) and upregulated (**F**) Reactome pathways in low *Fuz* expression group. (**G**) Protein-protein interaction analysis showed the interaction between upregulated genes (in diamond shape) and downregulated genes (in circular shape) from low *Fuz* expression group. The increasing degree of nodes was indicated by continuous color (light green-dark green). The size of nodes represents fold changes of gene expression. The densely connected network was isolated using MCODE function from Cytoscape. Genes from MCODE component were annotated for GO terms with DAVID v6.8.

All primary tumor tissues with available patient survival information (n = 498) were divided into high *Fuz* expression group (n = 409) and low *Fuz* expression group (n = 89) according to the survival probabilities of HNSC patients. The low *Fuz* expression group showed poor OS in HNSC (Figure 3A). To gain further insight into the gene expression features in the low *Fuz* expression group, differential gene expression analysis was performed between low *Fuz* expression and high *Fuz* expression groups (Supplementary File 1). The log_2_FC and adjusted *p* < 0.001 were used as selection criteria to determine upregulated (787 genes) and downregulated (4,748 genes) gene sets (Figure 3B). We then investigated whether these dysregulated genes are enriched in certain signaling pathways. GSEAPreranked analysis was performed to determine significantly enriched GO terms and Reactome pathways. Compared to the enriched GO terms (15 items) in the downregulated gene set, more (26 items) were found in the upregulated gene set (Figure 3C, 3D). Meanwhile, the Reactome pathway analysis demonstrated 5 enriched downregulated pathways and 27 enriched upregulated pathways (Figure 3E, 3F). We further selected the top 500 dysregulated genes based on the |log_2_FC| to construct a protein-protein interaction (PPI) network by using the STRING database (https://string-db.org/). The network was visualized using Cytoscape and the densely connected MCODE network was isolated. The MCODE network was consisted of 16 genes, which were found enriched in muscle development, organization and contraction (Figure 3G).

### Gene set enrichment analysis of genes from LUAD tumor samples with low *Fuz* expression

**Figure 4:**
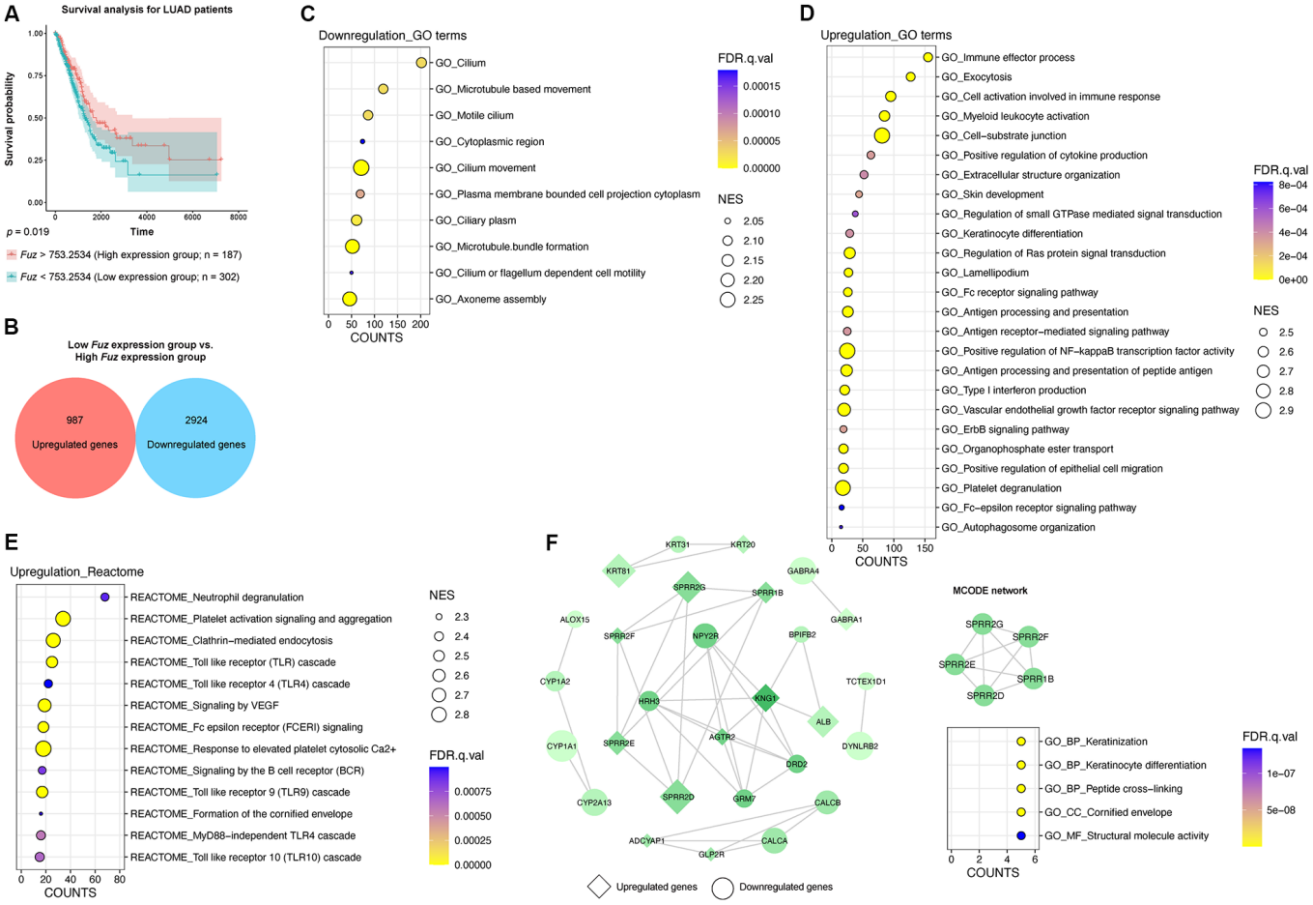
**The gene set enrichment and PPI analyses of dysregulated genes from LUAD tumor samples with low Fuz expression.** (**A**) The LUAD primary tumor tissues were divided into high *Fuz* expression and low *Fuz* expression groups based on the patient survival probabilities. (**B**) The number of upregulated and downregulated genes were calculated from LUAD primary tumor tissues with low *Fuz* expression. (**C**, **D**) The GO enrichment analysis demonstrated downregulated (**C**) and upregulated (**D**) genes-associated gene ontology terms in low *Fuz* expression group. (**E**) The Reactome pathway enrichment analysis demonstrated the upregulated Reactome pathways in low *Fuz* expression group. (**F**) Protein-protein interaction analysis showed the interaction between upregulated genes (in diamond shape) and downregulated genes (in circular shape) from low *Fuz* expression group. The increasing degree of nodes was indicated by continuous color (light green-dark green). The size of nodes represents fold changes of gene expression. The densely connected network was isolated using MCODE function from Cytoscape. Genes from MCODE component were annotated for GO terms with DAVID v6.8.

Similar to HNSC tumor samples (Figure 3A), we divided all LUAD primary tumor tissues with available patient survival information (n = 489) into high *Fuz* expression group (n = 187) and low *Fuz* expression group (n = 302). Poor OS in LUAD patients was observed in low *Fuz* expression group (Figure 4A). We then performed differential gene expression analysis between low and high *Fuz* expression groups (Supplementary File 1). The log_2_FC and adjusted *p* < 0.001 were used to select upregulated (987 genes) and downregulated (2,924 genes) gene sets in the low *Fuz* expression group (Figure 4B). The ranked gene set was submitted to GSEAPreranked for the subsequent enrichment analysis. Similar to what was observed in HNSC samples, more GO terms were enriched in upregulated gene set (25 items) compared to the downregulated one (10 items) (Figure 4C, 4D). The Reactome pathway analysis identified 13 enriched upregulated pathways (Figure 4E). The top 500 dysregulated genes were selected based on the |log_2_FC| for the following PPI network construction. The obtained PPI network from STRING database was visualized in Cytoscape software. The isolated MCODE network is composed of small proline-rich protein 1B (SPRR1B), small proline-rich protein 2D (SPRR2D), small proline-rich protein 2E (SPRR2E), small proline-rich protein 2F (SPRR2F) and small proline-rich protein 2G (SPRR2G), which are involved in Keratinization and Peptide cross-linking (Figure 4F).

### *Fuz* promoter methylation is responsible for *Fuz* transcriptional downregulation in ESCA, HNSC and LUAD tumor samples

**Figure 5:**
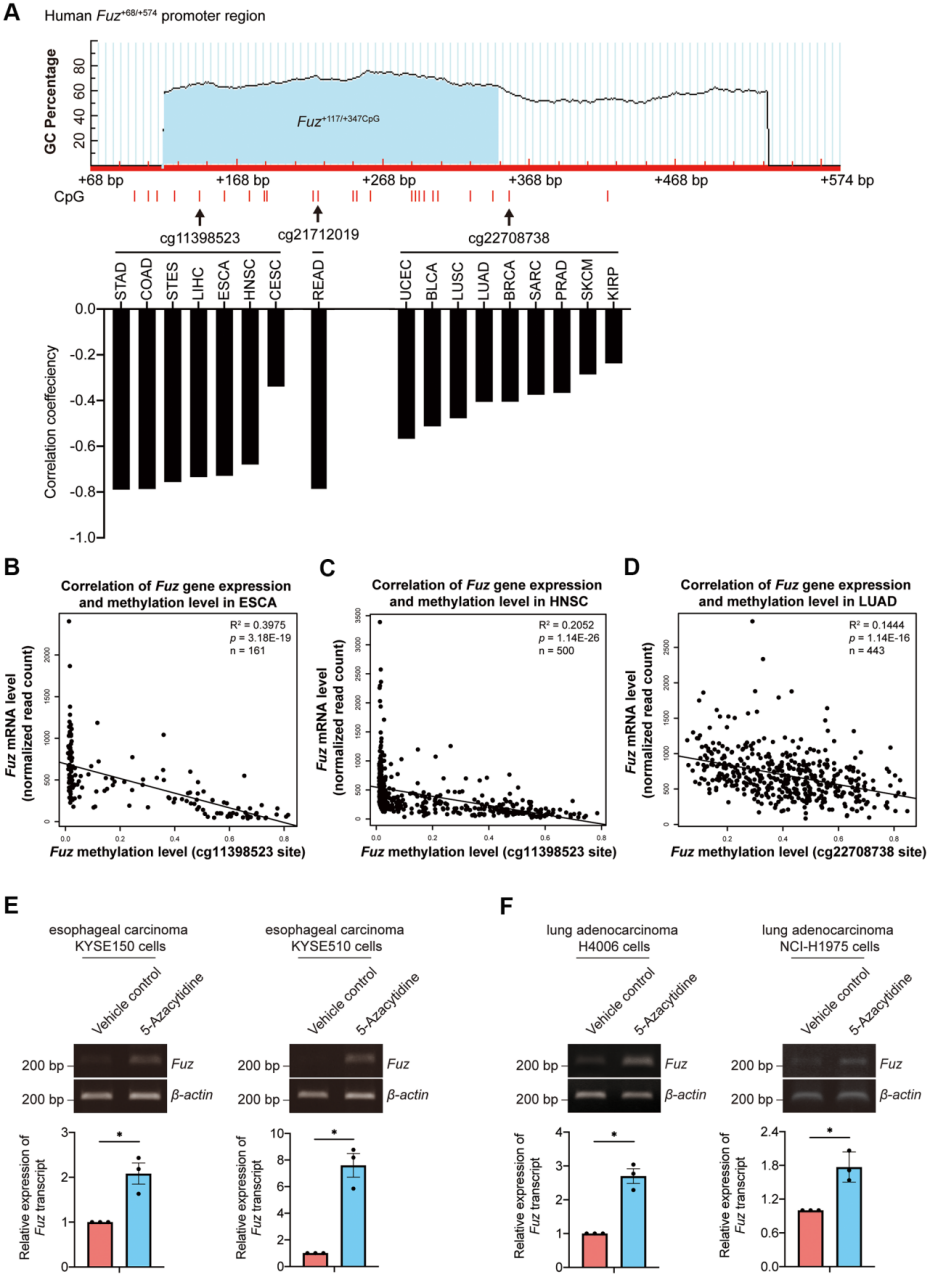
**Fuz expression was associated with its promoter methylation level in ESCA and LUAD tumor samples.** (**A**) *Fuz* methylation data obtained from Firebrowse database indicates that *Fuz* mRNA level was negatively associated with its promoter methylation level in various types of cancer. Three methylation sites (cg11398523, cg21712019 and cg22708738) were highlighted, and these sites reside within or close to a potential CpG island (*Fuz*^+117/+347CpG^) in *Fuz*^+68/+574^ promoter region. The CpG island *Fuz*^+117/+347CpG^ was predicted using MethPrimer software (https://www.urogene.org/cgi-bin/methprimer/methprimer.cgi) [27]. (**B**, **C**) *Fuz* mRNA level negatively correlates with the methylation at cg11398523 site within *Fuz* promoter in ESCA (**B**) and HNSC (**C**) patient samples. (**D**) A negative correlation between *Fuz* expression and *Fuz* promoter methylation level (at cg22708738 site) was identified in LUAD patient samples. (**E**) Treatment of 5-Azacytidine upregulated *Fuz* transcript level in esophageal carcinoma KYSE150 and KYSE510 cell lines. (**F**) *Fuz* transcript level was increased upon treatment of 5-Azacytidine in lung adenocarcinoma H4006 and NCI-H1975 cell lines. *n* = 3 biological replicates. Each *n* represents an independent preparation of cell RNA samples. Error bars represent S.E.M.. Statistical analysis was performed using two-tailed unpaired Student’s t-test. * denotes *p* < 0.05.

DNA methylation is one of the essential epigenetic regulators of gene expression [25]. Given that DNA methylation in gene promoter negatively controls gene transcription, we investigated whether the aberrant *Fuz* transcription is associated with alteration of *Fuz* promoter methylation in different types of cancer (Supplementary File 2). As shown in Figure 5A, 3 independent CpG methylation sites, cg11398523, cg21712019 and cg22708738, were identified within or close to a predicted CpG island in the *Fuz* promoter (*Fuz*^+117/+347CpG^) [23]. The DNA methylation levels at sites cg11398523 and cg22708738 were found negatively associated with *Fuz* expression in multiple types of cancer, including ESCA, HNSC and LUAD (Figure 5A). The mRNA expression and DNA methylation data obtained from TCGA further confirmed an inverse correlation between *Fuz* expression and its DNA methylation level in ESCA, HNSC and LUAD primary tumor tissues (Figure 5B–5D). Moreover, upon treatment of 5-Azacytidine, inhibitor of DNA methyltransferase, *Fuz* expression was upregulated in cell lines from esophageal carcinoma (Figure 5E) and lung adenocarcinoma (Figure 5F).

**Figure 6:**
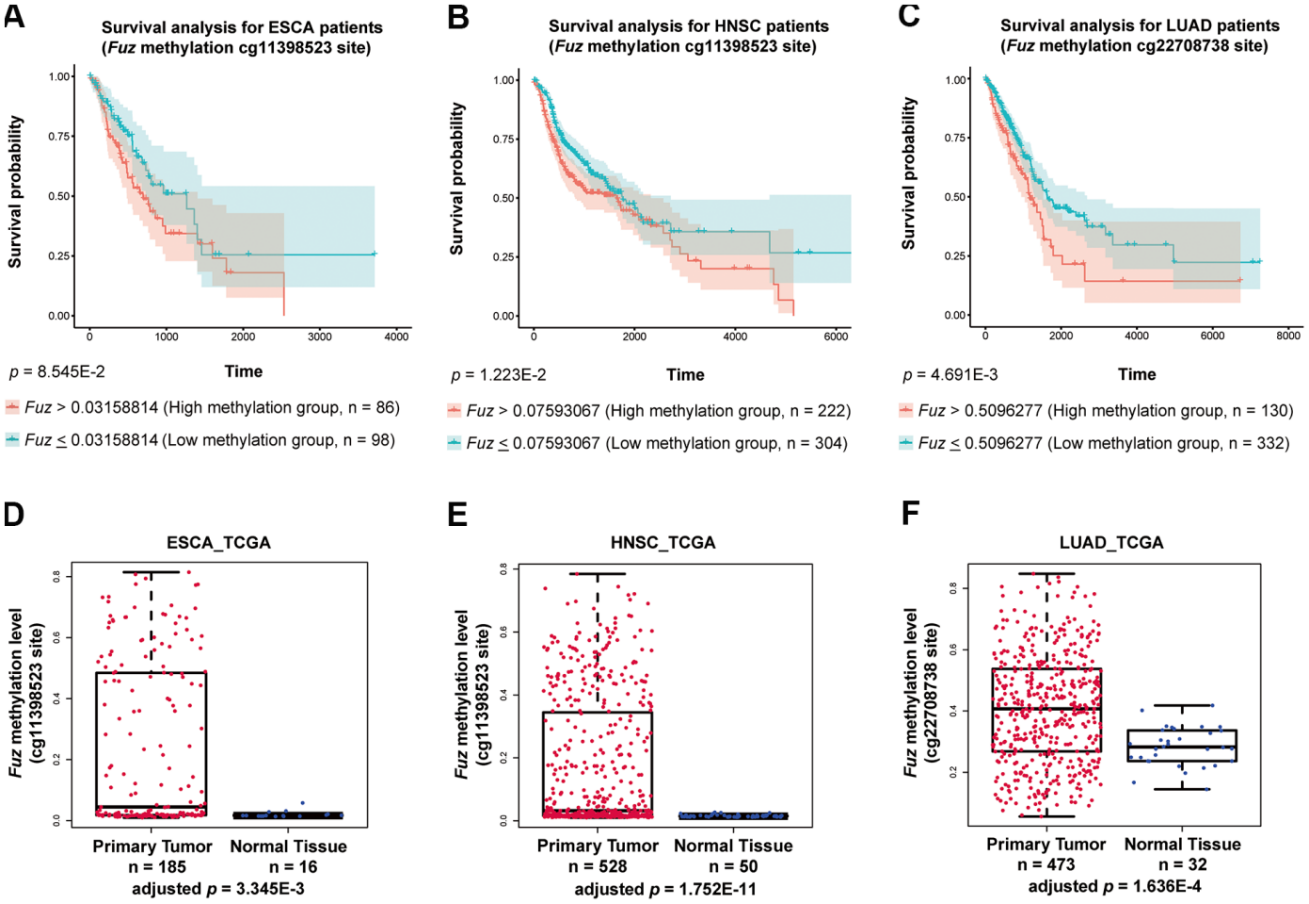
**High methylation of Fuz promoter associated with poor survival probabilities in HNSC and LUAD patients, and Fuz promoter methylation level was significantly upregulated in tumor samples from ESCA, HNSC and LUAD patients.** (**A**) Difference in *Fuz* promoter methylation level did not cause a significant alteration of survival probability in ESCA patients. (**B**, **C**) High *Fuz* promoter methylation leads to poor overall survival in HNSC (**B**) and LUAD (**C**) patients. (**D**, **E**) The methylation level of *Fuz* at cg11398523 site was significantly upregulated in ESCA (**D**) and HNSC (**E**) patient tumor samples. (**F**) The methylation level of *Fuz* at cg22708738 site was significantly upregulated in LUAD patient tumor samples.

We found that high *Fuz* DNA methylation at site cg11398523 and cg22708738 leads to reduced survival probabilities in HNSC and LUAD patients, respectively (Figure 6B, 6C). These coincide with our findings that lowered *Fuz* expression contributes to poor OS in HNSC and LUAD patients (Figures 3A, 4A). Meanwhile, lowered expression of *Fuz* was found associated with poor OS in ESCA patients (Supplementary Figure 4). Although a tendency of high *Fuz* DNA methylation level at site cg11398523 was observed in ESCA patients with poor OS, the difference in survival probabilities between high and low *Fuz* DNA methylation groups was not statistically significant (Figure 6A). We further compared *Fuz* DNA methylation levels between ESCA, HNSC and LUAD primary tumor tissues and their respective normal controls. As shown in Figure 6D–6F, a significant upregulation of *Fuz* DNA methylation level was detected in ESCA, HNSC and LUAD tumor samples. Taken together, these findings demonstrate a negative correlation between *Fuz* mRNA expression and *Fuz* DNA methylation level, and further suggest that *Fuz* promoter hypermethylation is a contributor to its transcriptional downregulation in ESCA, HNSC and LUAD.

### Identification of *Fuz* coding sequence alterations in multiple cancer types

In addition to gene dysregulation, coding sequence alteration is another pathogenic hallmark of cancer [26]. The mutant gene product generated may confer gain- or loss-of-function and affect outputs of cancer-related pathways.

**Figure 7:**
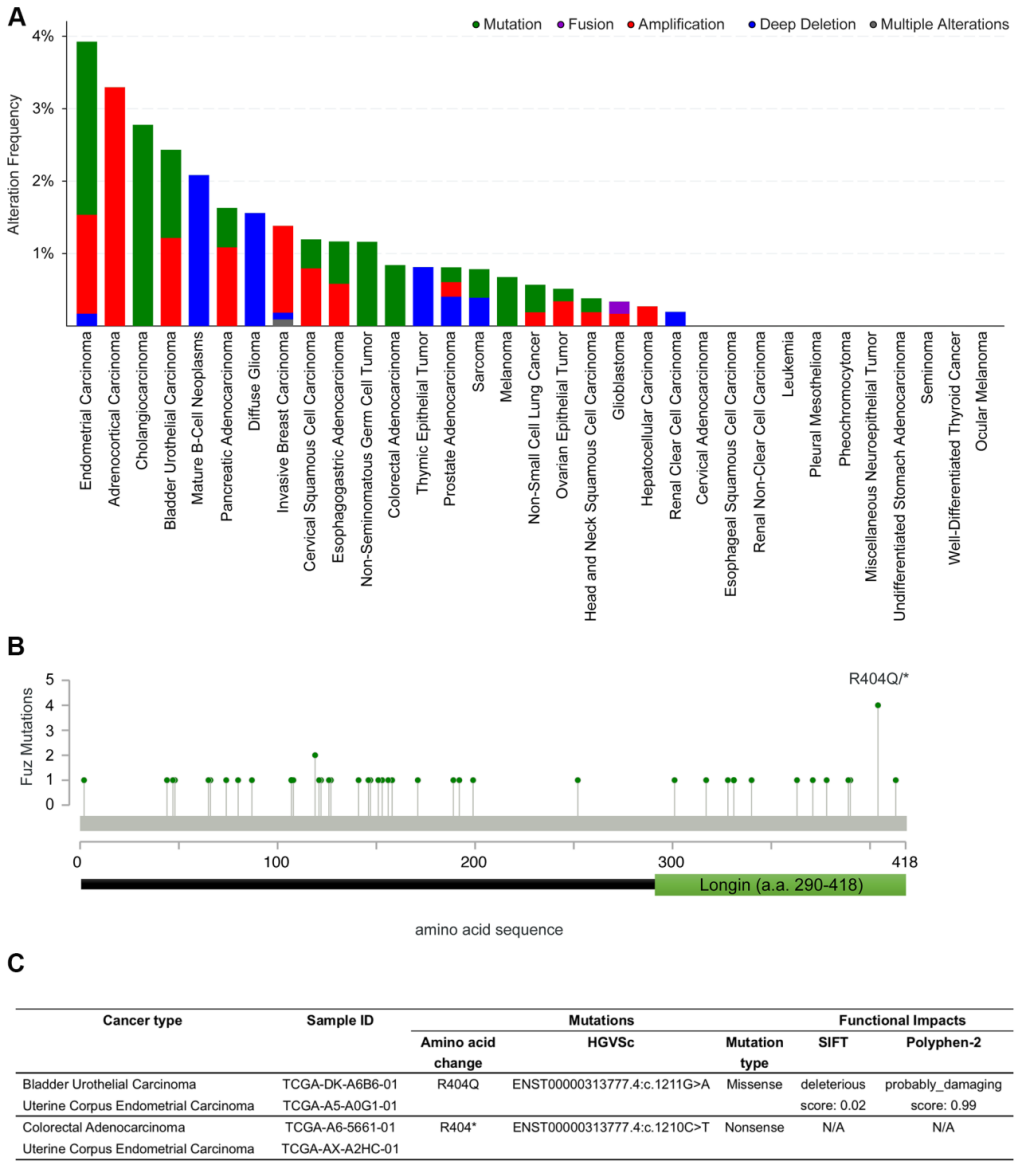
**Coding sequence alterations in Fuz were identified across multiple cancer types.** (**A**) cBioportal online database was used to investigate *Fuz* coding sequence alterations in multiples types of cancer. (**B**) A relative higher mutation frequency was identified at the 404^th^ arginine residue within the coding sequence at *Fuz* locus. (**C**) The missense and nonsense mutations at Fuz^R404^ were identified in 4 patient samples from 3 independent studies.

We thus explored if potential coding region variations in the *Fuz* gene exist in cancer genomes. The alteration frequency of *Fuz* was examined across 32 independent TCGA PanCancer Atlas Studies using the cBioPortal database (https://www.cbioportal.org/). Several kinds of genetic alterations, including mutation, fusion, amplification and deep deletion in the *Fuz* gene were uncovered from various cancer types, with the highest alteration frequency found in endometrial carcinoma samples (Figure 7A, Supplementary Table 2). Interestingly, a relatively higher mutation frequency at the 404^th^ amino acid position of the Fuz protein (Fuz^R404^) was identified (Figure 7B). The Fuz^R404^ arginine residue was found mutated in 4 tumor samples from bladder urothelial carcinoma, colorectal adenocarcinoma and uterine corpus endometrial carcinoma, and all these 4 mutated samples were caused by the single nucleotide substitution (Figure 7C). However, the missense mutation (c.G1211A) leads to a replacement of the arginine with glutamine (p.R404Q), whereas the nonsense mutation (c.C1210T) generates a truncated gene product (p.R404*; Figure 5C).

### Investigation of the functional consequence of mutant Fuz protein

We previously showed a novel pro-apoptotic function of Fuz protein, however, whether mutant Fuz^R404Q^ would affect the activation of cell apoptotic pathway remains elusive. We thus examined the pro-apoptotic property of mutant Fuz protein.

**Figure 8:**
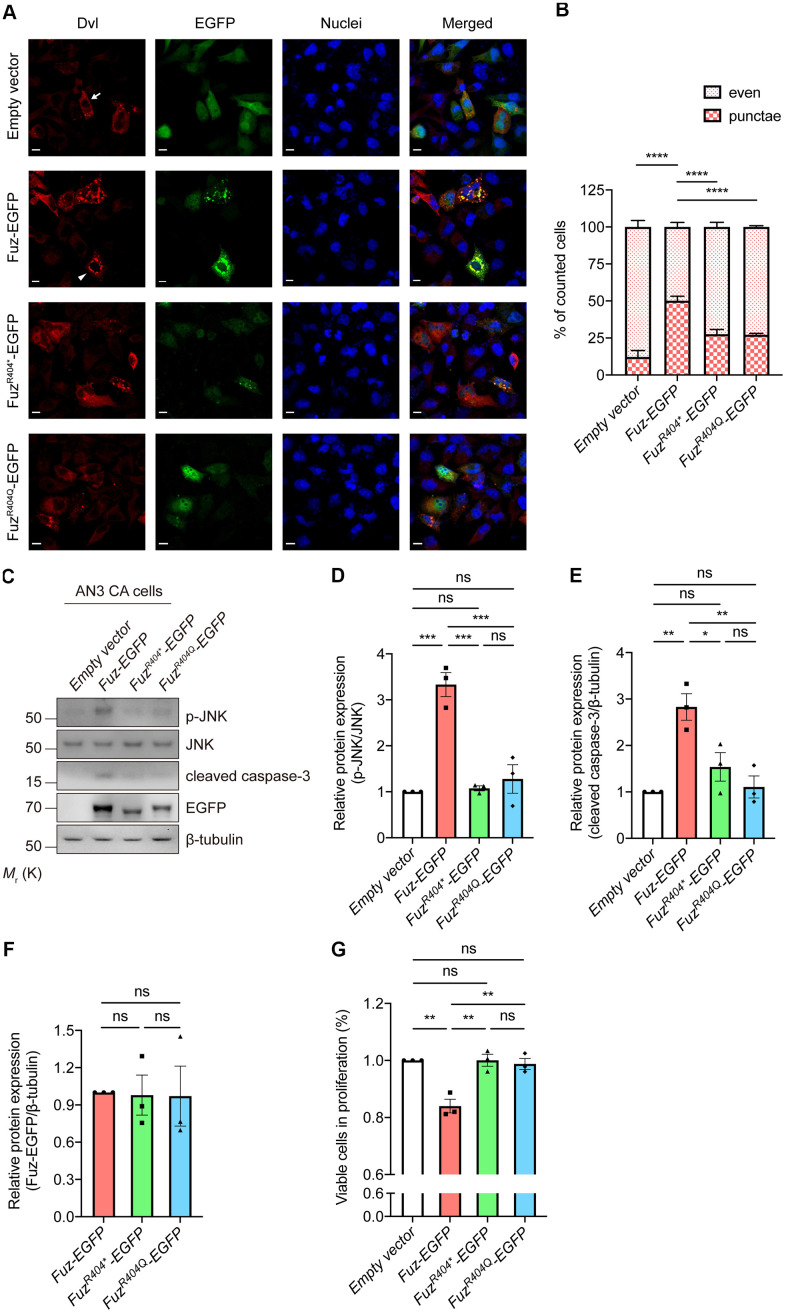
**Overexpression of mutant Fuz proteins did not lead to the activation of cell apoptotic pathway.** (**A**) When solely expressed in HEK293 cells, Dvl protein (red) showed two distinct staining patterns, which consist evenly distributed Dvl (arrow) and punctate Dvl (arrowhead). The evenly distributed Dvl is the predominant staining pattern. Overexpression of Fuz (green) promoted the formation of Dvl punctae, while such effect was attenuated in Fuz^R404*^ or Fuz^R404Q^-expressing cells. Cell nuclei (blue) were stained with Hoechst 33342. Scale bars: 10 μm. *n* = 3 biological replicates. Each *n* represents an independent preparation of immunocytochemistry sample. At least 100 cells were counted in each control or experimental group from an independent experiment. (**B**) is the quantification of (**A**). Error bars represent S.E.M. Statistical analysis was performed using one-way ANOVA followed by *post hoc* Tukey’s test. **** denotes *p* < 0.0001. (**C**) Overexpression of Fuz, but not Fuz^R404*^ or Fuz^R404Q^, promoted JNK-caspase-3 activation in HEK293 cells. *n* = 3 biological replicates. Each *n* represents an independent preparation of cell protein samples. (**D**–**F**) Quantification of p-JNK, cleaved caspase-3 and Fuz-EGFP protein expression in (**C**). Error bars represent S.E.M. Statistical analysis was performed using one-way ANOVA followed by *post hoc* Tukey’s test. ns denotes no significant difference, * denotes *p* < 0.05, ** denotes *p* < 0.01 and *** denotes *p* < 0.001. (**G**) Overexpression of Fuz, but not Fuz^R404*^ or Fuz^R404Q^, suppressed the percentage of viable cells in proliferation. *n* = 3 biological replicates. Each *n* represents an independent preparation of cell proliferation assay samples. Error bars represent S.E.M. Statistical analysis was performed using one-way ANOVA followed by *post hoc* Tukey’s test. ns denotes no significant difference, ** denotes *p* < 0.01.

The mutant Fuz expression construct harboring R404* or R404Q mutation was generated (Supplementary Figure 5). Fuz was reported to trigger cell apoptosis via activating Dvl-Rac1-MAPK-caspase-3 signaling axis [23]. We then tested the activity of this apoptotic pathway in Fuz^R404*^ or Fuz^R404Q^-expressing cells. When overexpressed in our HEK293 cell model, the wildtype Fuz protein activates Dvl protein aggregation (from 12.22% to 50.17%), whereas such activation was attenuated in Fuz^R404*^ (27.57%) or Fuz^R404Q^ (27.18%) overexpression cells (Figure 8A, 8B). Moreover, although expressed at comparable levels, the JNK-caspase-3 activation triggered by wildtype Fuz protein was found mitigated in Fuz^R404*^ or Fuz^R404Q^-expressing cells (Figure 8C–8F). In addition, the Fuz-mediated suppression of cell proliferation was alleviated in Fuz^R404*^ or Fuz^R404Q^ overexpression cells (Figure 8G). Taken together, these functional experiments suggest that mutation at 404^th^ arginine residue perturbs the biological function of Fuz in triggering apoptosis.

## DISCUSSION

Fuz is one of the PCP effectors that have been implicated in governing mammalian embryonic development [10, 21]. In addition to Fuz, Inturned and WDPCP are the other two PCP effector players. Unlike Inturned and WDPCP, which have been linked to different types of cancer [28–30], the study of Fuz in cancer remains under-investigated. In the current study, we uncovered that *Fuz* expression associates with patient survival probabilities in 8 types of cancer (Figure 1). In HNSC and LUAD, *Fuz* expression is reduced (Figure 2), and such reduction correlates with the poor survival probabilities in patients (Figures 3A, 4A). Interestingly, physical interactions have been demonstrated among PCP effectors, and their subcellular localization can be mutually modulated in *Drosophila* wing cells [9, 31]. Further investigations to consider Fuz, Inturned and WDPCP as a functional group could unveil more pathogenic details of PCP effectors in cancer.

Head and neck cancer and lung cancer are both the leading causes of cancer-associated mortality worldwide [32, 33]. As one of the key pathogenic features, gene dysregulation leads to dysfunctions of essential signaling pathways, which in turn contribute to cancer development and progression [34]. In this study, a GSEA approach was exploited to investigate enriched gene functions and pathways in HNSC and LUAD patients with poor OS (Figures 3, 4). In HNSC, the genes related to muscle contraction and immunoglobulin production and circulation were downregulated, whereas the upregulated genes were enriched in RNA processing and protein synthesis and modification (Figure 3C, 3D). Meanwhile, the Reactome pathway analysis highlights the enrichment of several cancer-related pathways, including mitotic metaphase and anaphase [35], cell cycle checkpoints [36] and transcriptional regulation by TP53 [37] (Figure 3F). Pharmacological inhibition of key factors related to cell cycle regulation, such as WEE1 G2 checkpoint kinase and checkpoint kinase 1, has been shown to force the HNSC cells skipping the cell cycle checkpoints, leading to accumulation of massive DNA damage and eventually cell death [38, 39]. Somatic mutation in TP53 is one of the most frequent genetic alterations among human cancers, including HNSC [40]. The variable TP53 mutational landscape in HNSC bears distinct activities of downstream pathological pathways, and mutant p53 dysregulates a range of oncogenic molecules in favor of malignant phenotype in HNSC cells [41–43]. In line with previous reports, our findings further emphasize the importance of cell cycle regulation and TP53 signaling in HNSC. In LUAD, the upregulated genes were enriched in immune response and exocytosis (Figure 4D). The Reactome pathway enrichment results further point out upregulation of several toll like receptor cascades, which are crucial for executing innate immune response (Figure 4E). Meanwhile, genes focusing on cilium organization were downregulated (Figure 4C). Interestingly, the histological examination of lung tumor samples reveals loss of ciliary structures, and the cilia-related gene expression was found markedly decreased [44, 45]. Moreover, stimulation of ciliogenesis combats against invasion, cell proliferation and epithelial-mesenchymal transition of lung cancer cells [46]. Given the role of PCP signaling in mediating ciliogenesis, our study further suggests the pathological significance of cilia biogenesis in LUAD.

*Fuz* transcription negatively correlates with its promoter DNA methylation level. When the human embryonic 293 cells are treated with 5-Azacytidine, a DNA methyltransferase inhibitor, *Fuz* mRNA level is found upregulated [23]. In our previous study, a putative CpG island within *Fuz* promoter, *Fuz*^+117/+347CpG^, was identified. We further reported that a transcriptional factor, YY1, maintains the methylation of *Fuz*^+117/+347CpG^ to achieve the transcriptional repression of *Fuz* expression [23]. In this study, we further showed that DNA methylation of three independent CpG sites negatively correlates with *Fuz* mRNA level (Figure 5A). All these three CpG sites reside within or close to *Fuz*^+117/+347CpG^, and two of them were found negatively associated with *Fuz* expression in various cancer types, including ESCA, HNSC and LUAD (Figure 5B–5D). Such association was further validated by experimental evidence. When the esophageal carcinoma and lung adenocarcinoma cells were treated with 5-Azacytidine, *Fuz* transcription was upregulated (Figure 5E, 5F). In 2017, Hao et al. [24] demonstrated a negative correlation between *Fuz* expression and its promoter DNA methylation level in LIHC tumor samples at another CpG site, cg19763319, which is also close to the *Fuz*^+117/+347CpG^ region. These findings highlight *Fuz*^+117/+347CpG^ as a core regulatory element in governing *Fuz* transcription. Any possible dysregulation of the *Fuz*^+117/+347CpG^ methylation level may contribute to the pathogenesis of human diseases, including cancer.

In this study, we also determined the functional consequence of mutant Fuz proteins, Fuz^R404*^ and Fuz^R404Q^. The mutation at Fuz^R404^ residue resides within a predicted Longin domain (a.a. 290-418) in the Fuz protein C-terminus [21, 31, 47]. When overexpressed in cells, the wildtype Fuz protein triggers cell apoptosis via activating Dvl-Rac1-MAPK-caspase signaling cascade [23]. However, such activation was found attenuated in Fuz^R404*^ or Fuz^R404Q^-expressing cells, suggesting the deterioration of Fuz pro-apoptotic function (Figure 8). Since Fuz^R404*^ and Fuz^R404Q^ mutations were identified in cancer patient samples, this functional evidence may provide more clues on how the perturbation of Fuz protein functions leads to the cell overproliferation in certain types of cancer.

Both R404* and R404Q mutations occur in the highly conserved 404^th^ arginine residue within the Fuz Longin domain [47]. The presence of Longin domain is broadly shared by vesicle trafficking proteins [48, 49]. These Longin domain-containing proteins transport specific cargos and target their membrane localization in support of cilia outgrowth, which is impaired when protein dysfunctions occur [11, 50]. Interestingly, the R404Q mutation was initially identified from human patients with neural tube defects, a severe neurological deficit due to the failure of neural tube closure during embryonic development, and Seo et al. [47] showed that Fuz^R404Q^ mutant prevents cilia elongation and directional cell movements. Taken together, these findings suggest the importance of Longin domain in carrying out Fuz biological function, especially in regulating ciliogenesis. In our functional experiments, we found that, both Fuz^R404*^ and Fuz^R404Q^ mutants alleviated the stimulation of cell apoptosis (Figure 8). It would therefore be intriguing to further investigate the functional significance of Longin domain with respect to the pro-apoptotic activity of Fuz protein.

In conclusion, our study is the first report to demonstrate the mechanistic insights of a PCP effector, *Fuz*, in multiple types of cancer. The development of interventions targeting *Fuz* DNA methylation alteration and coding sequence mutations may be therapeutically beneficial towards carcinogenesis.

## MATERIALS AND METHODS

### Kaplan-Meier plotter analysis

The pan-cancer survey from the online database Kaplan-Meier Plotter (https://kmplot.com/analysis/index.php?p=service&cancer=pancancer_rnaseq) was used to evaluate the prognostic value of *Fuz* mRNA expression in 21 different types of cancer [51]. In particular, the prognostic value of *Fuz* mRNA expression in different breast cancer subtypes was analyzed separately (https://kmplot.com/analysis/index.php?p=service&cancer=breast). Moreover, the prognostic value of *Fuz* mRNA expression in additional Gene Expression Omnibus datasets of breast cancer (https://kmplot.com/analysis/index.php?p=service&cancer=breast), lung cancer (https://kmplot.com/analysis/index.php?p=service&cancer=lung) and gastric cancer (https://kmplot.com/analysis/index.php?p=service&cancer=gastric) was also assessed using Kaplan-Meier Plotter. The patient samples were split into two groups (high expression *vs*. low expression) based on the auto selected best cutoff for *Fuz* expression. The overall survival probability of cancer patients was assessed using the Kaplan-Meier survival plots, and logrank *p* value was calculated to determine whether the association between *Fuz* expression and patient survival is statistically significant.

### Processing of TCGA data

The RNA sequencing data and DNA methylation data of primary tumor tissues and solid normal tissues were obtained from The Cancer Genome Atlas (TCGA) Research Network (https://www.cancer.gov/tcga) via the GDC Data Portal (https://portal.gdc.cancer.gov/) [52] on June 27, 2018. The raw gene expression counts generated by HTSeq [53] were imported to DESeq2 (1.24.0) [54] in R (v3.6.3) for normalization and the normalized gene counts were used for downstream analysis. DESeq2 was used to perform the differential gene expression analysis, and standard DESeq2 Wald test, followed by the Benjamini-Hochberg correction was used for multiple comparisons. Wilcoxon Rank Sum test followed by Benjamini-Hochberg correction was used to compare methylation levels between tumor and normal tissues. To determine the correlation between *Fuz* mRNA level and *Fuz* DNA methylation level, the normalized gene counts were regressed on the DNA methylation levels using linear model with the function ‘lm’ in R. The Cox regression was performed using the ‘survival’ (3.2.7) and ‘survminer’ (0.4.7) packages in R to determine the association between *Fuz* DNA methylation level and patient survival probabilities. Multivariate survival analysis was performed again, with ‘survival’ and survminer’, using the seven factors retrieved from the TCGA clinical data, including gender, race, ethnicity, primary_diagnosis, tumor stage and age at diagnosis. In addition, the normalized expression level of *Fuz* was also included.

### Gene set enrichment analysis (GSEA)

The gene enrichment analysis was carried out using GSEA v4.0.3 software (https://www.gsea-msigdb.org/gsea/index.jsp) [55]. The gene ranks were generated by using DESeq2 to calculate fold changes of normalized gene counts between low *Fuz* and high *Fuz* expression groups in HNSC and LUAD tumor samples (Supplementary File 1). The annotated gene sets c5.go.v7.2.symbols.gmt was used for the Gene Ontology (GO) terms enrichment analysis, while the annotated gene set c2.cp.reactome.v7.2.symbols.gmt was used for the Reactome pathways enrichment analysis. The GSEAPreranked analysis was performed with the number of permutations set as 1,000 times for each analysis. The selection criteria for significantly enriched GO terms and Reactome pathways were |normalized enrichment score (NES)| > 2 and false discovery rate (FDR) *q*-value < 0.001.

### Protein-protein interaction (PPI) network analysis

The PPI network analysis was performed using STRING v11.0 database (https://string-db.org/) [56]. The protein-protein interaction network was constructed based on experimental evidence, computational predictions and co-expression networks. Top 500 dysregulated genes selected based on |log_2_FC| from low *Fuz* expression groups were used as input, and the minimum required interaction score was defined as highest confidence (0.900) to carry out the prediction. The Cytoscape v3.8.0 was used to visualize the constructed PPI network, and Molecular Complex Detection (MCODE) algorithm was used to select densely connected networks. The significantly (FDR < 0.001) enriched GO terms in the MCODE networks were isolated from DAVID 6.8 database (https://david.ncifcrf.gov/home.jsp) [57].

### cBioPortal analysis

cBioPortal v3.3.5 is a comprehensive web resource to provide visualization and analysis of cancer genomic data (https://www.cbioportal.org/) [58]. The genomic alteration profiles in *Fuz*, including mutations and copy number alterations, were obtained from 10,967 samples of 32 independent TCGA PanCancer Atlas Studies.

### Molecular cloning

The *pcDNA3.1 (zeo)-flag-Dvl* was a kind gift from Prof. Randall Moon (Addgene plasmid # 16758). The *Fuz-EGFP* was described previously [23]. The *Fuz*^R404*^ DNA sequence was amplified from *Fuz-EGFP* using primers *EcoRI-Fuz-F*, 5`-CCGGAATTCATGGGGGAGGAGGGGAC-3` and *KpnI-Fuz*^R404 stop^*-R*, 5`-CCGGGTACCGTTCACAGCCCATGGGTG-3`. The resultant DNA fragment was subcloned into *pEGFP-N1* (Clontech Laboratories) expression vector using *Eco*RI and *Kpn*I to generate *Fuz*^R404*^-EGFP mutant construct. Overlapping PCR method was used to generate the *Fuz*^R404Q^ mutant sequence, the resultant DNA fragment was subcloned into *pEGFP-N1* expression vector using *Eco*RI and *Kpn*I to generate *Fuz*^R404Q^*-EGFP* mutant construct. Primers used for overlapping PCR were *EcoRI-Fuz-F*, 5`-CCGGAATTCATGGGGGAGGAGGGGAC-3`, *Fuz*^R404Q^*-F*, 5`- ACCCATGGGCTGCAAAGCCTGGCC-3`, *Fuz*^R404Q^*-R*, 5`- GGCCAGGCTTTGCAGCCCATGGGT-3` and *KpnI-Fuz-R*, 5`- CCGGGTACCGTAAGAAGTGGGGTGAGG-3`.

### Cell culture and plasmid transfection

The human esophageal carcinoma cell lines KYSE150 and KYSE510 were kind gifts from Prof. Qian Tao (Department of Clinical Oncology, The Chinese University of Hong Kong) [59]. The human endometrial adenocarcinoma cell line AN3 CA was a kind gift from Prof. Chi Chiu Wang (Department of Obstetrics and Gynecology, The Chinese University of Hong Kong). The human lung adenocarcinoma cell lines H4006 (CRL-2871™) and NCI-H1975 (CRL-5908™) were obtained from American Type Culture Collection. All cell lines were cultured using Gibco™ RPMI 1640 Medium (21875034, Thermo Fisher Scientific) supplemented with 10% fetal bovine serum (F7524, Sigma-Aldrich) and 1% Antibiotic-Antimycotic solution (15240062, Thermo Fisher Scientific). The cells were maintained in a 37° C humidified cell culture incubator supplemented with 5% CO_2_. Lipofectamine 2000 (11668019, Thermo Fisher Scientific) was used in plasmid transfection. The ratio between plasmid (μg) and Lipofectamine 2000 (μl) was 1:2. For immunocytochemistry samples preparation, 0.3 μg *pcDNA3.1 (zeo)-flag-Dvl*, together with 0.5 μg *EGFP-N1*, *Fuz-EGFP*, *Fuz*^R404*^-EGFP or *Fuz*^R404Q^*-EGFP*, were used for transfection. The transfection lasted for 48 h. For immunoblotting samples preparation, 1.0 μg *EGFP-N1*, *Fuz-EGFP*, *Fuz*^R404*^-EGFP or *Fuz*^R404Q^*-EGFP* was used for transfection, and the transfection lasted for 72 h. For cell proliferation assay, 0.3 μg *EGFP-N1*, *Fuz-EGFP*, *Fuz*^R404*^-EGFP or *Fuz*^R404Q^*-EGFP* was used for transfection, and the transfection lasted for 72 h.

### Drug treatment

The KYSE150, KYSE510, H4006 and NCI-H1975 cells were treated with 2 μM 5-Azacytidine (0210082150, MP Biomedicals™). The treatment lasted 72 h, with medium and drug refreshed every 24 h.

### Reverse transcription PCR

RNAs were extracted from cancer cell lines using the Qiagen RNeasy Mini Kit (74104, Qiagen). One microgram of RNA was used for reverse transcription using ImProm-II^TM^ Reverse Transcription System (A3800, Promega), according to the manufacturer’s instructions. Primers used in this study were *Human Fuz-119-F*, 5`-TCTCTGTCATCGGTTCCCTC-3`; *Human Fuz-366-R*, 5`-CTCCACGTTGCGGATATTGG-3`; *Actin-F*, 5`-ATGTGCAAGGCCGGTTTCGC-3`; *Actin-R*, 5`-CGACACGCAGCTCATTGTAG-3`. The PCR products were amplified using Phusion™ High-Fidelity DNA Polymerase (F530S, Thermo Fisher Scientific) and visualized on the Bio-Rad ChemiDoc imaging system.

### Immunocytochemistry

The AN3 CA cells were seeded on coverslips (Marienfeld-Superior). After 48 h, cells were transfected. After another 48 h, transfected cells were fixed with 3.7% paraformaldehyde for 15 min followed by permeabilization with 0.1% Triton X-100 for another 15 min. The cells were blocked with 5% goat serum at 25° C for 1 h, followed by the incubation with primary antibody at 4° C for 16 h. The cells were then washed 3 times with 1X PBS for 5 min each. The secondary antibody was used to incubate cells at 25° C for 1 h. The cells were then washed 5 times with 1X PBS for 5 min each. The primary and secondary antibodies used were anti-flag (1:200; F3165, Sigma-Aldrich) and goat anti-mouse IgG (H+L) Cy3 conjugate (1:400; 81-6515, Zymed, Thermo Fisher Scientific). The cell nuclei were stained with Hoechst 33342 (1:400; H-1399, Thermo Fisher Scientific) at 25° C for 5 min. Cell images were acquired using a confocal microscope Zeiss LSM (Zeiss) and images were analyzed using Fiji software (Version 2.0.0-rc-69/1.52n, NIH).

### Immunoblotting

The AN3 CA cells were seeded in 24-well plates (3526, Corning). After 48 h, cells were transfected. After another 72 h, transfected cells were harvested in SDS sample buffer (100 mM Tris-HCl, pH 6.8, 2% SDS, 40% glycerol, 5% β-mercaptoethanol and 0.1% bromophenol blue). Samples were heated at 99° C for 10 min prior to being subjected to the immunoblot analysis. Primary antibodies used were anti-p-JNK (1:500, 9251, Cell Signaling Technology), anti-JNK (1:1,000, 9252, Cell Signaling Technology), anti-cleaved caspase-3 (1:500; 9664, Cell Signaling Technology), anti-GFP (1:2,000; 632381, Clontech Laboratories, Inc.) and anti-β-tubulin (1:2,000; ab6046, Abcam). Secondary antibodies used for immunoblotting were HRP conjugated Goat anti-Rabbit IgG (H+L) (1:5,000, G-21234, Thermo Fisher scientific) and HRP conjugated Goat anti-Mouse IgG (H+L) (1:5,000, G-21040, Thermo Fisher Scientific). The signal was developed using Immobilon Forte Western HRP substrate (WBLUF0100, Merck Millipore) and visualized on the Bio-Rad ChemiDoc imaging system.

### Cell proliferation assay

The AN3 CA cells were seeded in 96-well plates (3603, Corning). After 48 h, cells were transfected. After another 72 h, transfected cells were added with CellTiter 96^®^ AQueous One Solution Reagent (G3582, Promega). The reaction was incubated at 37° C for 1 h, followed by the measurement of absorbance at 490 nm on a FLUOstar Omega Microplate Reader (BMG LABTECH).

## Supplementary Material

Supplementary Figures

Supplementary Tables

Supplementary File 1

Supplementary File 2
