# Supplementary material for: Pan-cancer investigation reveals mechanistic insights of planar cell polarity gene Fuz in carcinogenesis
Source: Aging (Albany NY). 2021 Feb 26;13(5):7259–83. doi: 10.18632/aging.202582 (PMC7993721; doi:10.18632/aging.202582)
Supplement: Supplementary Figures [file aging-13-202582-s001.pdf]

SUPPLEMENTARY FIGURES

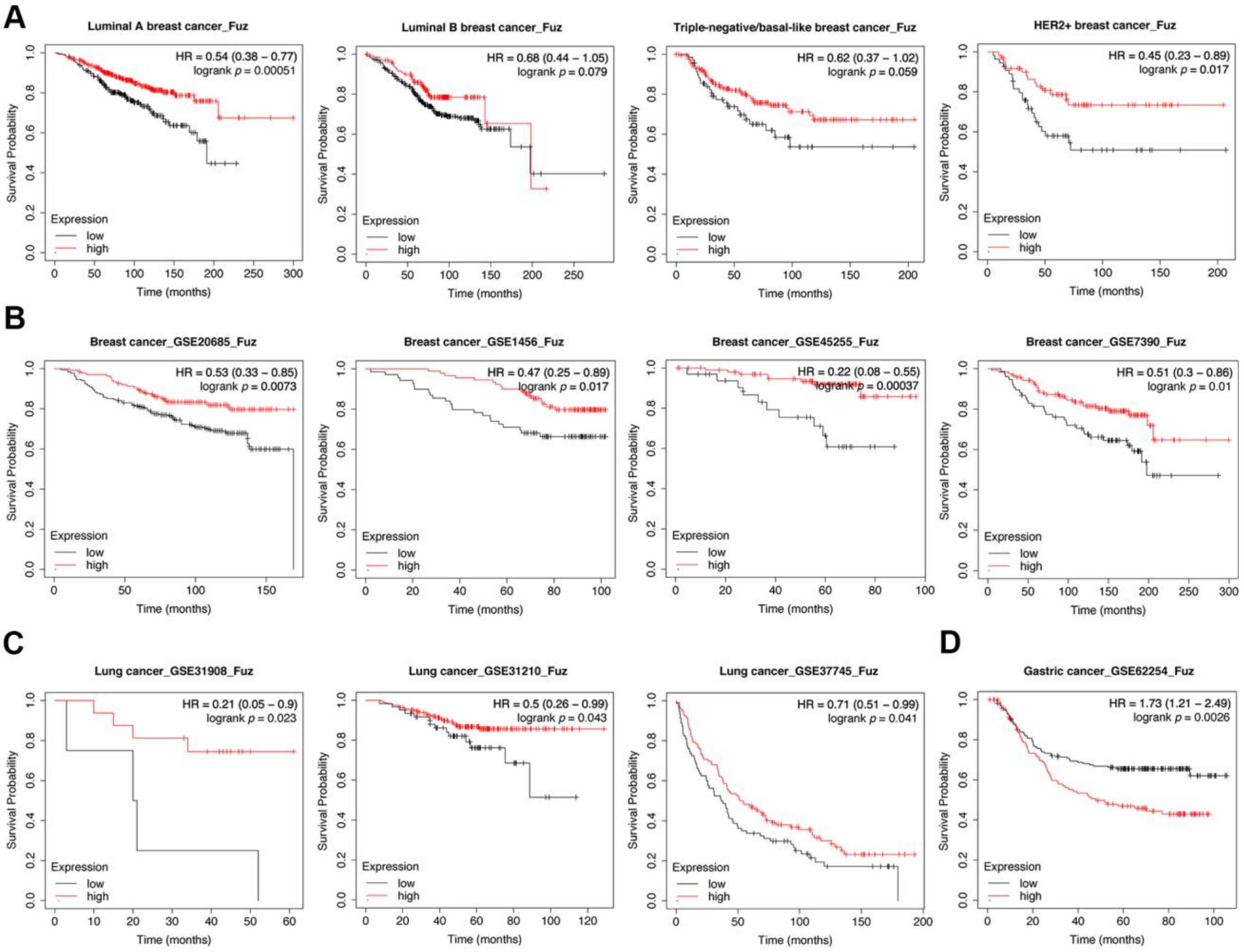

Supplementary Figure 1. Evaluation of the prognostic significance of *Fuz* mRNA expression in different subtypes of breast cancer and different cancer types. (**A**) Low level of *Fuz* expression correlated with poor prognosis in luminal A and HER2+ breast cancer patients, whilst no significant correlation was found in luminal B and triple-negative/basal-like breast cancer patients. (**B**–**D**) The correlation between low *Fuz* expression and poor prognosis of breast cancer (**B**), lung cancer (**C**) and gastric cancer (**D**) patients was further validated using additional GEO datasets.

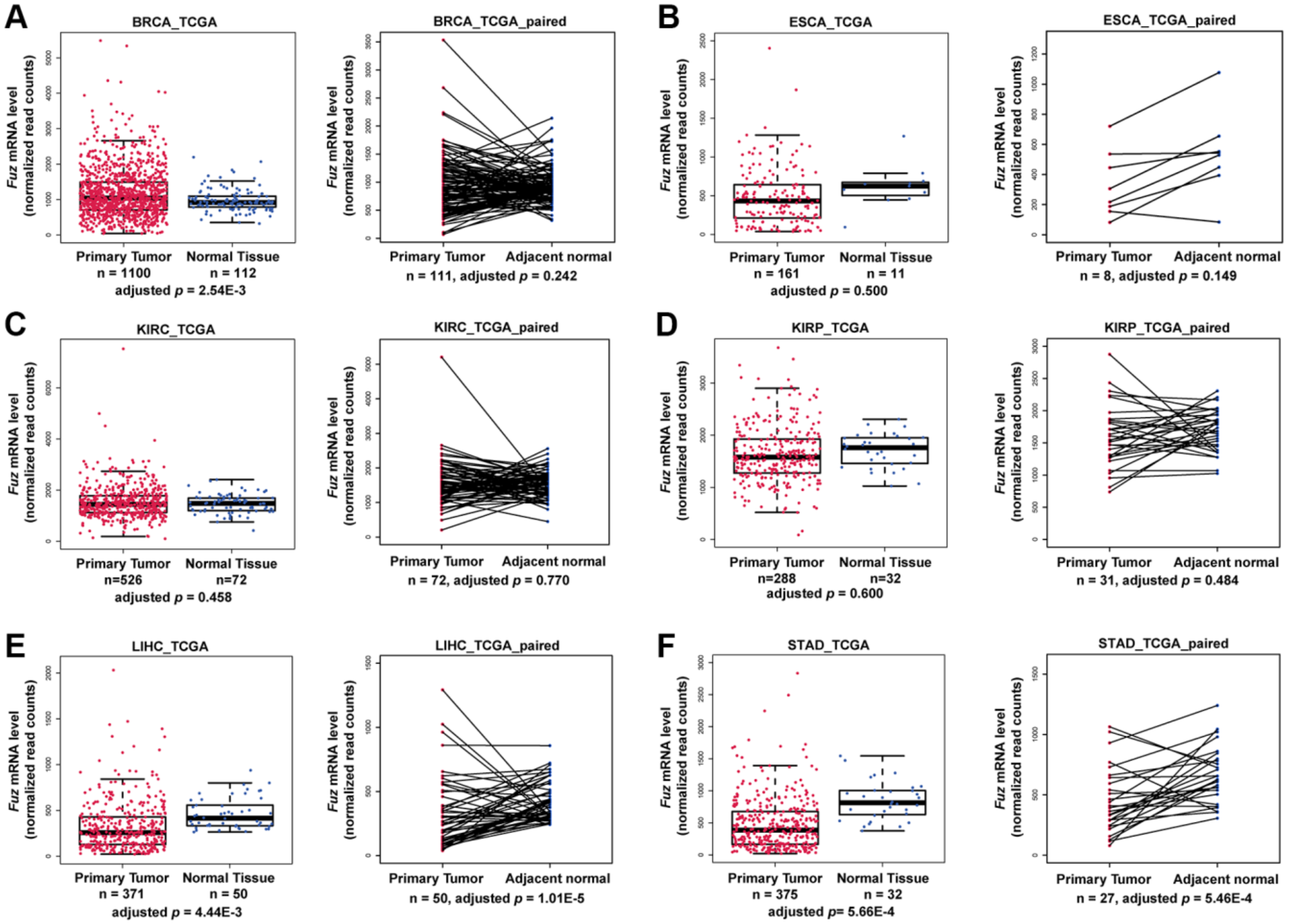

Supplementary Figure 2. Investigation of Fuz transcript level in different types of cancer. (**A**–**F**) Significant changes in Fuz mRNA level was found in tumor tissues from BRCA (**A**), LIHC (**E**) and STAD (**F**) patients. Downregulation of Fuz was further found in paired samples from LIHC (**E**) and STAD (**F**) patients. No significant changes in Fuz mRNA level were identified in tumor tissues from ESCA (**B**), KIRC (**C**) and KIRP (**D**) patients.

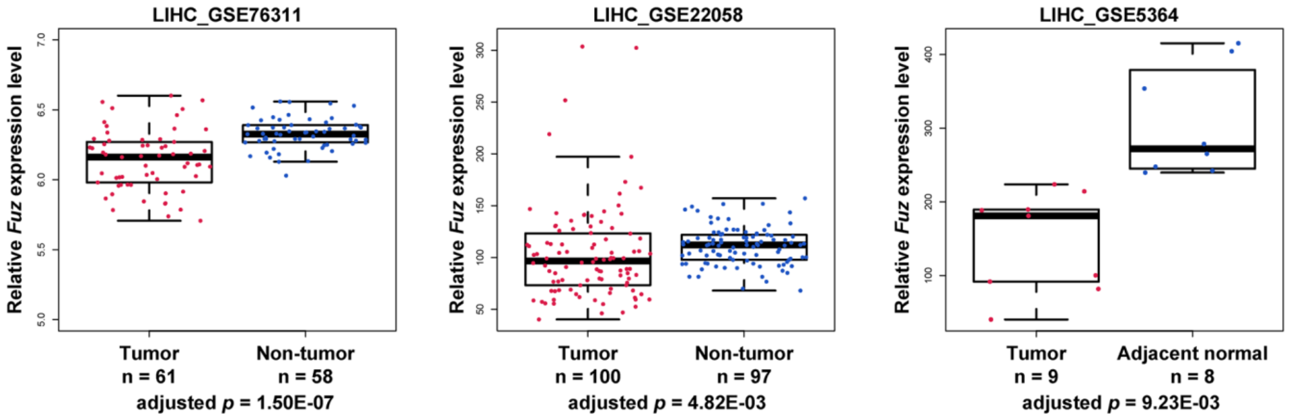

Supplementary Figure 3. Downregulation of Fuz expression level was further validated in additional LIHC datasets of GSE76311, GSE22058 and GSE5364.

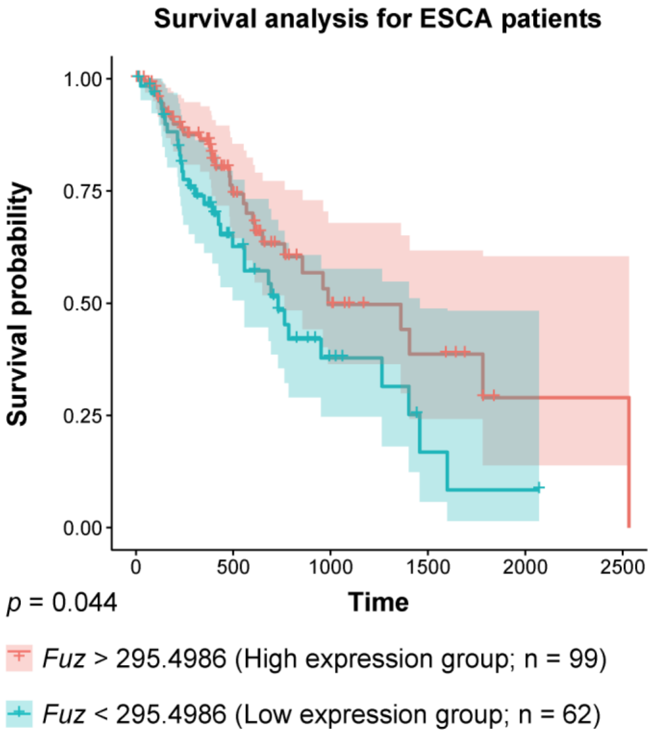

Supplementary Figure 4. The ESCA primary tumor tissues were divided into high *Fuz* expression and low *Fuz* expression groups based on the patient survival probabilities. Low *Fuz* expression associated with poor survival probabilities from ESCA patients.

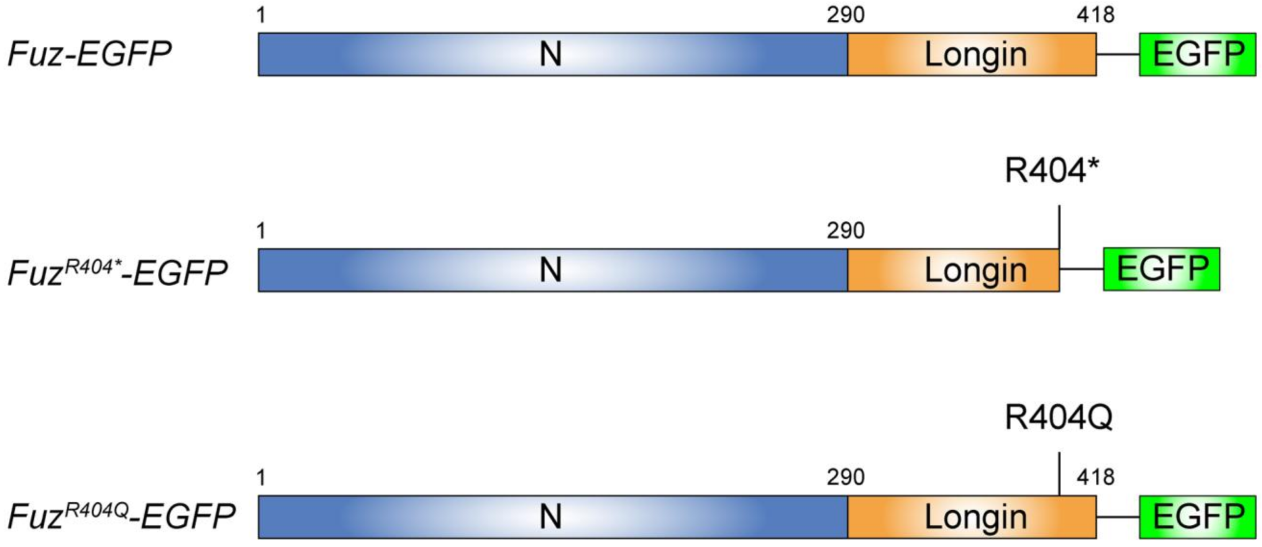

Supplementary Figure 5. Wildtype and mutant Fuz constructs used in the functional experiments. The location of R404* and R404Q mutations are shown.
