# Supplementary material for: Pan-cancer investigation reveals mechanistic insights of planar cell polarity gene Fuz in carcinogenesis
Source: Aging (Albany NY). 2021 Feb 26;13(5):7259–83. doi: 10.18632/aging.202582 (PMC7993721; doi:10.18632/aging.202582)
Supplement: Supplementary Tables [file aging-13-202582-s002.pdf]

## SUPPLEMENTARY TABLES

**Supplementary Table 1. Multivariate analysis of correlation of different disease parameters in BRCA, ESCA, HNSC, KIRC, KIRP, LIHC, LUAD or STAD patients.**

| Parameter | Multivariate analysis_BRCA | | | Multivariate analysis_ESCA | | |
|---|---|---|---|---|---|---|
| | HR | 95% CI | *p* value | HR | 95% CI | *p* value |
| Gender | 0.9537 | 0.1292 - 7.042 | 0.9630 | 1.247 | 0.0927 - 16.766 | 0.8679 |
| Race | 0.893 | 0.6298 - 1.266 | 0.5250 | 0.725 | 0.3132 - 1.678 | 0.45264 |
| Ethnicity | 5.8278 | 0.8107 - 41.895 | 0.0799 | 1.469 | 0.1216 - 17.765 | 0.76212 |
| Primary diagnosis | 0.7095 | 0.5378 - 0.936 | 0.0152 | 1.904 | 0.6730 - 5.384 | 0.22494 |
| Tumor stage | 1.2114 | 1.1428 - 1.284 | 1.10E-10 | 1.525 | 1.1233 - 2.071 | 0.00682 |
| Age at diagnosis | 1.0001 | 1.0001 - 1.000 | 1.28E-08 | 1.000 | 0.9999 - 1.000 | 0.40249 |
| *Fuz* level | 0.9996 | 0.9993 – 1.000 | 0.0106 | 0.998 | 0.9957 - 1.000 | 0.09167 |
| **Parameter** | **Multivariate analysis_HNSC** | | | **Multivariate analysis_KIRC** | | |
| | **HR** | **95% CI** | ***p* value** | **HR** | **95% CI** | ***p* value** |
| Gender | 0.8247 | 0.5842 - 1.164 | 0.27342 | 0.9170 | 0.6353 - 1.324 | 0.64357 |
| Race | 0.8430 | 0.6128 - 1.160 | 0.29424 | 0.9811 | 0.5549 - 1.735 | 0.94774 |
| Ethnicity | 0.9505 | 0.4698 - 1.923 | 0.88771 | 4.8638 | 1.5412 - 15.350 | 0.00698 |
| Primary diagnosis | 0.9786 | 0.9571 - 1.001 | 0.05591 | N/A | N/A | N/A |
| Tumor stage | 1.5944 | 1.3186 - 1.928 | 1.48E-06 | 1.8836 | 1.6176 - 2.193 | 3.56E-16 |
| Age at diagnosis | 1.0001 | 1.0000 - 1.000 | 0.00296 | 1.0001 | 1.0000 - 1.000 | 0.00224 |
| *Fuz* level | 0.9995 | 0.9989 - 1.000 | 0.10457 | 1.0000 | 0.9997 - 1.000 | 0.93238 |

| Parameter | Multivariate analysis_KIRP | | | Multivariate analysis_LIHC | | |
|---|---|---|---|---|---|---|
| | HR | 95% CI | *p* value | HR | 95% CI | *p* value |
| Gender | 0.798 | 0.3419 - 1.863 | 0.60189 | 0.8888 | 0.5772 - 1.369 | 0.593 |
| Race | 0.805 | 0.3225 - 2.010 | 0.64221 | 0.9669 | 0.7706 - 1.213 | 0.771 |
| Ethnicity | 2.1369 | 0.2799 - 16.317 | 0.46407 | 1.2104 | 0.4700 - 3.117 | 0.692 |
| Primary diagnosis | N/A | N/A | N/A | N/A | N/A | N/A |
| Tumor stage | 2.9383 | 2.0429 - 4.226 | 6.18E-09 | 1.2998 | 1.1749 - 1.438 | 3.64E-07 |
| Age at diagnosis | 1.0001 | 1.0000 - 1.000 | 0.06171 | 1.0000 | 1.0000 - 1.000 | 0.369 |
| *Fuz* level | 0.9988 | 0.9981 – 1.000 | 0.00241 | 1.0001 | 0.9994 - 1.001 | 0.825 |
| **Parameter** | **Multivariate analysis_LUAD** | | | **Multivariate analysis_STAD** | | |
| | **HR** | **95% CI** | ***p* value** | **HR** | **95% CI** | ***p* value** |
| Gender | 0.9682 | 0.6656 - 1.408 | 0.866 | 1.071 | 0.6869 - 1.670 | 0.76191 |
| Race | 1.2936 | 0.8047 - 2.080 | 0.288 | 1.052 | 0.8713 - 1.270 | 0.59922 |
| Ethnicity | 1.1651 | 0.2779 - 4.885 | 0.834 | N/A | N/A | N/A |
| Primary diagnosis | 0.9688 | 0.8138 - 1.153 | 0.721 | 9.533 | 0.7760 - 1.171 | 0.64897 |
| Tumor stage | 1.2151 | 1.1197 - 1.319 | 3.04E-06 | 1.181 | 1.0738 - 1.300 | 6.24E-04 |
| Age at diagnosis | 1.0000 | 1.0000 - 1.000 | 0.414 | 1.000 | 1.0000 - 1.000 | 0.02021 |
| *Fuz* level | 1.0001 | 0.9996 - 1.001 | 0.593 | 1.001 | 1.0002 - 1.001 | 0.00257 |

**Supplementary Table 2. The detailed number of cases with *Fuz* genomic alterations in different types of cancer.**

| Cancer types | Total cases | Altered cases | | | | |
|---|---|---|---|---|---|---|
| | | Mutation | Amplification | Deep deletion | Multiple alterations | Fusion |
| Endometrial Carcinoma | 586 | 14 (2.39%) | 8 (1.37%) | 1 (0.17%) | 0 | 0 |
| Adrenocortical Carcinoma | 91 | 0 | 3 (3.3%) | 0 | 0 | 0 |
| Cholangiocarcinoma | 36 | 1 (2.78%) | 0 | 0 | 0 | 0 |
| Bladder Urothelial Carcinoma | 411 | 5 (1.22%) | 5 (1.22%) | 0 | 0 | 0 |
| Mature B-Cell Neoplasms | 48 | 0 | 0 | 1 (2.08%) | 0 | 0 |
| Pancreatic Adenocarcinoma | 184 | 1 (0.54%) | 2 (1.09%) | 0 | 0 | 0 |
| Diffuse Glioma | 513 | 0 | 0 | 8 (1.56%) | 0 | 0 |
| Invasive Breast Carcinoma | 1084 | 0 | 13 (1.2%) | 1 (0.09%) | 1 (0.09%) | 0 |
| Cervical Squamous Cell Carcinoma | 251 | 1 (0.4%) | 2 (0.8%) | 0 | 0 | 0 |
| Esophagogastric Adenocarcinoma | 514 | 3 (0.58%) | 3 (0.58%) | 0 | 0 | 0 |
| Non-Seminomatous Germ Cell Tumor | 86 | 1 (1.16%) | 0 | 0 | 0 | 0 |
| Colorectal Adenocarcinoma | 594 | 5 (0.84%) | 0 | 0 | 0 | 0 |
| Thymic Epithelial Tumor | 123 | 0 | 0 | 1 (0.81%) | 0 | 0 |
| Prostate Adenocarcinoma | 494 | 1 (0.2%) | 1 (0.2%) | 2 (0.4%) | 0 | 0 |
| Sarcoma | 255 | 1 (0.39%) | 0 | 1 (0.39%) | 0 | 0 |
| Melanoma | 444 | 3 (0.68%) | 0 | 0 | 0 | 0 |
| Non-Small Cell Lung Cancer | 1053 | 4 (0.38%) | 2 (0.19%) | 0 | 0 | 0 |
| Ovarian Epithelial Tumor | 584 | 1 (0.17%) | 2 (0.34%) | 0 | 0 | 0 |
| Head and Neck Squamous Cell Carcinoma | 523 | 1 (0.19%) | 1 (0.19%) | 0 | 0 | 0 |
| Glioblastoma | 592 | 0 | 1 (0.17%) | 0 | 0 | 1 (0.17%) |
| Hepatocellular Carcinoma | 369 | 0 | 1 (0.27%) | 0 | 0 | 0 |
| Renal Clear Cell Carcinoma | 511 | 0 | 0 | 1 (0.2%) | 0 | 0 |
